# Comparative Evaluation of Sputum and Bronchoalveolar Lavage (BAL) Cultures in Clinically Suspected Cases of Community-Acquired Lower Respiratory Tract Infections

**DOI:** 10.7759/cureus.103400

**Published:** 2026-02-11

**Authors:** Sonali Maheshwari, Vrushali Patwardhan, Tulika Choudhary, Vikas Dogra, Shikhar Saxena

**Affiliations:** 1 Microbiology, Institute of Human Behaviour and Allied Sciences, New Delhi, IND; 2 Microbiology, Post Graduate Institute of Child Health, Noida, IND; 3 Microbiology, Rama Medical College Hospital and Research Centre, Hapur, IND; 4 Pulmonology, Rajiv Gandhi Super Speciality Hospital, Delhi, IND; 5 Microbiology, Rajiv Gandhi Super Speciality Hospital, Delhi, IND

**Keywords:** bronchoalveolar lavage (bal), clinically suspected, diagnostic efficacy, lower respiratory tract infection, sputum

## Abstract

Objectives: This study aimed to compare diagnostic agreement between culture results from bronchoalveolar lavage (BAL) and sputum samples for common pneumonia-causing pathogens and to evaluate the value of less invasive alternative diagnostic methods for pneumonia.

Materials and methods: This retrospective study was conducted at a tertiary care hospital in North India from January 2022 to December 2022. Samples were inoculated directly onto blood agar, chocolate agar, and MacConkey agar. Confirmation of isolates and antimicrobial susceptibility testing was performed using an automated system (VITEK 2, bioMérieux, Marcy-l’Étoile, France). A total of 253 paired BAL and sputum samples collected in close succession were analyzed for concordance. Statistical analyses were performed using SPSS Statistics version 28 (IBM Corp. Released 2021. IBM SPSS Statistics for Windows, Version 28.0. Armonk, NY: IBM Corp.). The intraclass correlation coefficient (ICC) was used to assess the level of agreement between the two methods.

Results: Among 253 patients, 171 (67.6%) were male, with a mean age of 56.7 ± 2.1 years. Significant bacterial growth was observed in 43 (16.9%) sputum and 55 (21.7%) BAL specimens. *Pseudomonas aeruginosa* was the most common isolate in both sample types. Overall concordance between sputum and BAL cultures was 80.2%, with near-perfect agreement for P. aeruginosa (ICC = 0.90). The sensitivity and specificity of sputum culture were 78.2% and 100%, respectively, compared with BAL.

Conclusions: Sputum culture serves as a reliable first-line diagnostic tool with high specificity, whereas BAL demonstrates superior sensitivity and pathogen yield. BAL should be considered in cases with negative or inconclusive sputum results to enhance diagnostic accuracy in lower respiratory tract infections.

## Introduction

Pneumonia is an infection of the lower respiratory tract that involves the lung parenchyma and remains a significant cause of morbidity and mortality worldwide. Community-acquired pneumonia (CAP) is defined as pneumonia acquired outside the hospital setting [1,2]. Globally, CAP affects millions of adults annually and is a leading cause of hospitalization, particularly among elderly individuals and those with underlying comorbidities. It represents a substantial proportion of infection-related mortality and continues to pose a significant public health burden despite advances in antimicrobial therapy and vaccination strategies [1,2]. Given the wide variety of causative agents, accurate and early detection of pneumonia remains an active area of research [1,3].

Early and precise diagnosis of pneumonia, as well as its differentiation from upper respiratory tract infections, is essential to avoid unnecessary antibiotic prescriptions. Rapid identification of the causative pathogen is crucial for minimizing diagnostic delays and ensuring the prompt use of appropriate antibiotics, particularly in an era of increasing antibiotic resistance and escalating healthcare costs [2]. CAP is associated with higher hospitalization rates and longer hospital stays, thereby increasing both the clinical and economic burden. Moreover, CAP has considerable long-term effects on patients’ quality of life even after apparent clinical recovery [2,4].

Determining the microbial cause of pneumonia remains challenging, as obtaining high-quality samples from the lower respiratory tract is often difficult, and blood cultures frequently yield negative results [5]. Several diagnostic modalities are currently employed, including blood cultures, urinary antigen testing, serological assays, and molecular techniques such as polymerase chain reaction (PCR); however, each method has inherent limitations related to sensitivity, specificity, cost, availability, and turnaround time. Despite the development of various techniques for collecting respiratory specimens, the causative pathogen is identified in only about 60-70% of pneumonia etiologic studies, with clinical practice reporting an even lower identification rate of around 30% [5,6].

Since the early 1900s, sputum culture has been used to identify pathogens in cases of pneumonia [5]. Because of its non-invasive nature, low cost, and ease of collection, sputum examination remains the most commonly utilized microbiological investigation in routine clinical practice for patients with suspected CAP [2]. Although it remains the first step in in-hospital analysis, its sensitivity, reliability, and influence on treatment decisions for different pathogens have been widely debated [2,7,8]. In addition to being difficult to obtain in some patients, sputum specimens are often contaminated with microorganisms from the upper respiratory tract, resulting in inconclusive results [9,10].

Fiber-optic bronchoscopy, particularly bronchoalveolar lavage (BAL), has emerged as a valuable method for identifying pathogens that do not typically colonize the upper respiratory tract. In this procedure, a bronchoscope is used to instill saline into the bronchioles and alveoli to obtain samples from distal airways [5,11,12]. BAL generally offers greater microbiological specificity and reduced contamination compared with sputum samples; however, its invasive nature, specialized expertise requirements, and variable diagnostic yield limit its routine application [13]. Nevertheless, the most recent clinical practice guidelines from the Infectious Diseases Society of America recommend obtaining lower respiratory tract specimens in patients with CAP who are immunocompromised, have failed treatment, or are suspected of having hospital-acquired or ventilator-associated pneumonia [2,4].

Given the widespread routine use of sputum culture and the more selective use of BAL in clinical practice, understanding the diagnostic agreement between these two methods is clinically relevant. This study aims to compare diagnostic agreement between culture results from BAL and sputum samples for the most common pneumonia-causing pathogens and to assess the diagnostic value of less invasive sampling methods. The clinical usefulness of sputum culture has been widely debated. Therefore, this retrospective analysis compares standard culture results from patients who underwent both BAL and sputum sampling to determine the positive predictive value of sputum culture relative to BAL fluid culture.

## Materials and methods

This retrospective, single-center observational study was conducted at a tertiary care hospital in North India from January 2022 to December 2022. Adult patients clinically suspected of having community-acquired acute lower respiratory tract infection (LRTI) were included. Clinical suspicion was based on acute respiratory symptoms, such as cough, fever, dyspnea, and/or purulent sputum, along with at least one systemic feature suggestive of infection. In contrast, radiological suspicion was established by chest radiography or computed tomography, which demonstrated new or progressive pulmonary infiltrates, consolidation, or ground-glass opacities consistent with LRTI. Patients were eligible for inclusion if both sputum and BAL specimens were collected as part of routine clinical care, with BAL performed within three days of sputum collection. To allow accurate paired comparison and minimize contamination-related bias, only cases yielding monomicrobial growth of Gram-negative bacteria in both specimen types were included. Patients in pediatric age groups, those without paired BAL specimens collected within three days of sputum sampling, and those with polymicrobial cultures were excluded. After applying these criteria, a total of 253 paired sputum and BAL samples obtained from 253 adult patients were included in the final analysis.

All sputum specimens were collected in the hospital environment as part of routine clinical practice, with patients instructed regarding proper sputum collection technique, and were transported immediately to the microbiology laboratory in sterile wide-mouthed containers. The quality of sputum samples was assessed using Bartlett’s grading system following Gram staining, and only specimens deemed satisfactory were processed further. BAL was performed using a standardized flexible fiber-optic bronchoscopy technique under local anesthesia and conscious sedation in accordance with American Thoracic Society guidelines [14]. The bronchoscope was advanced into segmental or subsegmental bronchi corresponding to radiologically involved lung segments, and sterile normal saline was instilled in 20-50 mL aliquots and gently aspirated to obtain samples from the distal airways. Recovered lavage fluid was transported immediately to the microbiology laboratory for processing.

Both sputum and BAL specimens were directly inoculated onto blood agar, chocolate agar, and MacConkey agar within two hours of collection. For BAL specimens, a positive quantitative culture was defined as ≥1 × 10⁴ CFU/mL. Identification of bacterial isolates and antimicrobial susceptibility testing were performed using an automated system (VITEK 2®, bioMérieux, Marcy-l’Étoile, France). Concordance and discordance between sputum and BAL culture results, including antimicrobial susceptibility profiles, were subsequently analyzed.

Statistical analysis

All data were compiled and entered into Microsoft Excel (Microsoft Corp., Redmond, WA, USA) and double-checked for accuracy. Categorical variables were expressed as absolute numbers and percentages. For diagnostic performance analysis, BAL culture results were used as the reference (gold) standard, and the sensitivity and specificity of sputum culture were calculated using standard 2 × 2 contingency tables, with corresponding 95% confidence intervals (CIs). Statistical significance was assessed using a two-sided test, and p-values <0.05 were considered significant.

To assess the level of agreement between paired sputum and BAL culture results, the intraclass correlation coefficient (ICC) was calculated with 95% CIs. A two-way mixed-effects model with an absolute agreement definition was used, as the same two diagnostic methods (sputum and BAL) were applied to all patients and were not considered interchangeable. ICC values were interpreted as follows: 0 indicating no agreement, 0.01-0.20 poor agreement, 0.21-0.40 fair agreement, 0.41-0.60 moderate agreement, 0.61-0.80 substantial agreement, 0.81-0.99 near-perfect agreement, and 1.00 perfect agreement. All statistical analyses were performed using SPSS Statistics version 28 (IBM Corp. Released 2021. IBM SPSS Statistics for Windows, Version 28.0. Armonk, NY: IBM Corp.).

## Results

**Figure 1:**
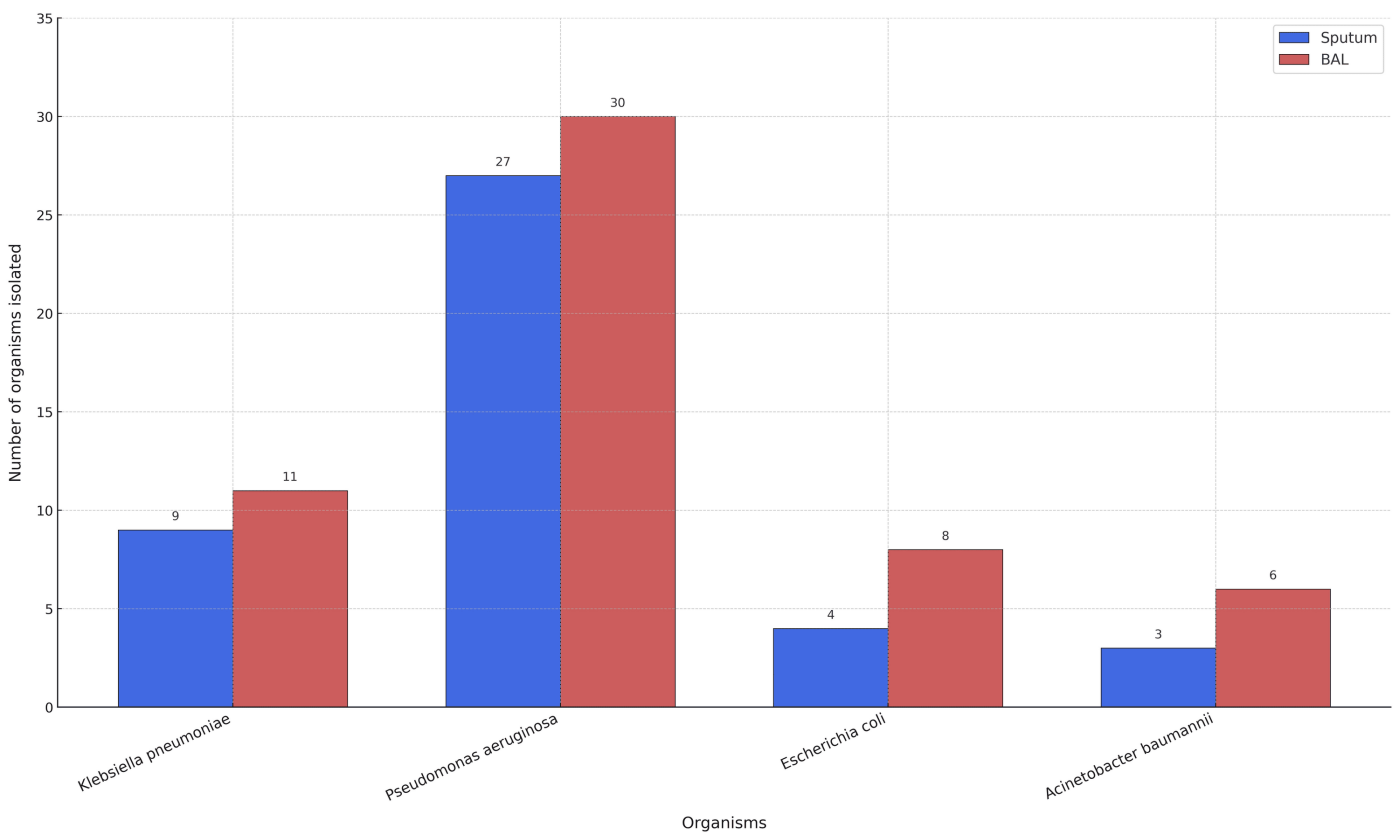
Graph representing various organism isolated from sputum and BAL sample BAL: bronchoalveolar lavage

The study population comprised 171 (67.6%) males and 82 (32.4%) females. The mean age was 56.7 ± 2.1 years for males and 41.4 ± 2.18 years for females. Significant bacterial growth was observed in 43 (16.9%) sputum samples, predominantly *Pseudomonas aeruginosa* (27, 62.8%), followed by *Klebsiella pneumoniae* (9, 20.9%), *Escherichia coli* (4, 9.3%), and *Acinetobacter baumannii* complex (3, 7.0%). BAL cultures yielded a higher positivity rate. Meanwhile, 55 (21.7%) samples showed significant growth, including all organisms detected in sputum. Overall, BAL identified 12 (4.7%) additional pathogen isolates compared with sputum culture (Figure 1).

Of 253 evaluated cases, sputum cultures were positive in 43 (17.0%), whereas BAL cultures identified organisms in 55 (21.7%), indicating a higher microbiological yield with BAL. The overall concordance between sputum and BAL was 80%, with good reliability (ICC = 0.80) and a positive percent agreement of 83.6%. *Pseudomonas aeruginosa* was the predominant isolate, recovered in 27 (10.7%) sputum samples and 30 (11.9%) BAL samples, with excellent concordance of 90% (ICC = 0.90). *Klebsiella pneumoniae* was detected in nine (3.6%) sputum and 11 (4.3%) BAL samples, with 80% concordance (ICC = 0.81). In contrast, *Escherichia coli* was identified in four (1.6%) sputum samples and eight (3.2%) BAL samples, whereas *Acinetobacter baumannii* was identified in three (1.2%) sputum samples and six (2.4%) BAL samples; both organisms showed 50% concordance (ICC = 0.50). These findings indicate that BAL provides superior overall organism detection, with particularly strong agreement for *Pseudomonas aeruginosa* and *Klebsiella pneumoniae*, whereas agreement for *Escherichia coli* and *Acinetobacter baumannii* was moderate (Table 1).

**Table 1:** Organism isolated from sputum and BAL sample BAL: bronchoalveolar lavage, ICC: intraclass correlation coefficient, CI: confidence interval

Organism	Sputum positive (n = 253)	Bronchoalveolar lavage (n = 253)	Concordant cases (%)	ICC (95% CI)	Positive percent agreement
Klebsiella pneumoniae	9	11	80%	0.81	84.60%
Pseudomonas aeruginosa	27	30	90%	0.9	90%
Escherichia coli	4	8	50%	0.5	66.70%
Acinetobacter baumannii	3	6	50%	0.5	66.70%
Total	43	55	80%	0.8	83.60%

The overall sensitivity and specificity of sputum culture were 78.2% and 100%, respectively, compared with BAL culture results (Table 2).

**Table 2:** Sensitivity and specificity of sputum culture compared to BAL specimens from 2 × 2 matrix of results TP: true positive, TN: true negative, FP: false positive, FN: false negative, BAL: bronchoalveolar lavage Sensitivity = TP/TP + FN​ × 100 = 80% and specificity = TN/TN+FP​ × 100 = 100%

Sputum culture	BAL culture (gold standard)		Total
	Positive	Negative	
Positive	43 (TP)	0 (FP)	43
Negative	12 (FN)	198 (TN)	210
Total	55	198	253

## Discussion

The results of our study demonstrate the diagnostic utility of both sputum and BAL specimens in identifying pathogenic microorganisms in patients with LRTI. The majority of patients in this study were male (67.6%), with a mean age of 56.7 ± 2.1 years, consistent with epidemiological trends in respiratory infections, in which males are more frequently affected [15,16].

Among the 253 sputum specimens collected, 16.9% demonstrated significant pathogen growth, with *Pseudomonas aeruginosa* being the most frequently isolated organism. In contrast, BAL specimens demonstrated a higher culture positivity rate (21.7%) than sputum samples. Similar findings have been reported in other studies comparing the diagnostic efficacy of these two specimen types [15].

The isolation of identical pathogens from both sputum and BAL samples reinforces that both specimen types provide comparable microbiological information about the respiratory flora. However, BAL detected 12 additional cases of significant pathogen growth, indicating a higher pathogen detection yield compared to sputum culture. This observation may reflect the advantage of BAL samples being collected directly from the lower respiratory tract, which provides a closer representation of the site of infection. However, the difference in pathogen recovery may also be partially influenced by the time interval between sputum and BAL sample collection, rather than solely reflecting intrinsic differences in test sensitivity.

The diagnostic role of sputum specimens in pneumonia has long been debated, with studies reporting varying levels of reliability. Sputum cultures are currently recommended primarily for CAP when results are expected to influence antibiotic selection [2]. However, in 2002, Ewig et al. [7] found that sputum cultures had limited diagnostic value and that their routine use may not significantly affect patient outcomes due to relatively low diagnostic yield.

Zhang et al. [8] advanced this comparison by analyzing the agreement between BAL and sputum cultures in children with suspected CAP. They found moderate agreement between the two specimen types, indicating partial overlap in pathogen detection, but also that each method may identify distinct pathogens or miss others altogether. Our findings are consistent with their observations, as we noted a significant concordance rate of 80% between sputum and BAL cultures. Nonetheless, BAL demonstrated higher sensitivity, identifying additional cases that sputum cultures failed to detect.

In this study, sputum and BAL cultures showed high concordance (80%), with near-perfect agreement for *Pseudomonas aeruginosa* (ICC = 0.9) and substantial agreement for *Klebsiella pneumoniae* (ICC = 0.81), indicating that sputum samples can reliably reflect BAL findings for these pathogens. Moderate agreement for *Escherichia coli* and *Acinetobacter baumannii* suggests that sputum may occasionally miss certain organisms. These findings are consistent with those of Dubong et al. [16], who reported 96.5% concordance and a high positive predictive value for sputum compared with BAL, particularly for common pathogens. Conversely, Zhang et al. [8] reported lower sensitivity and poor specificity of sputum Gram stains in predicting BAL pathogens, highlighting the limitations of sputum in detecting all organisms. Collectively, these results suggest that while sputum is a useful, less invasive first-line sample, BAL remains the gold standard for comprehensive pathogen detection, especially for less prevalent or fastidious bacteria.

The sensitivity of sputum culture, compared with BAL (80%), was moderate but clinically relevant, indicating that sputum culture can detect most pathogens identified by BAL. However, it may fail to identify cases with lower bacterial loads or infections localized to the deeper lung regions. The perfect specificity (100%) of sputum culture in this study demonstrates that when a pathogen is detected in sputum, it is almost always accurate, with no false positives relative to BAL results. These findings align with the published literature, which reports comparable conclusions [8].

Our results have important clinical implications, particularly in healthcare settings with limited resources, where patient tolerance, cost, or laboratory capacity may restrict the routine use of BAL. In such settings, sputum culture remains a practical and valuable first-line tool for pathogen detection, as supported by our high concordance rates for *Pseudomonas aeruginosa* and *Klebsiella pneumoniae*. These findings are consistent with those of Dubong et al. [16], who reported greater than 96% concordance between sputum and BAL cultures, underscoring the reliability of sputum cultures for detecting common pathogens. However, moderate agreement for organisms such as *Escherichia coli* and *Acinetobacter baumannii* in our study aligns with Zhang et al., who reported lower sensitivity and specificity of sputum in predicting BAL pathogens, emphasizing that sputum may occasionally miss certain infections. Therefore, when sputum cultures are negative despite strong clinical suspicion or when infections involve deeper pulmonary structures, BAL provides a more comprehensive assessment and remains the gold standard for accurate pathogen identification. These findings underscore the importance of optimizing diagnostic strategies to match available resources while ensuring patient safety and diagnostic accuracy.

Limitations

One limitation of this study is its retrospective design, which may have introduced selection bias and limited control over variables such as the timing of sample collection and antibiotic exposure. The exclusive reliance on culture-based methods for pathogen identification is another limitation, as molecular diagnostic techniques, such as PCR and NGS, can provide faster, more sensitive detection of pathogens, including those difficult to culture. Additionally, this study included only specimens with monomicrobial growth of Gram-negative bacteria, which may limit the generalizability of the findings. There was a time gap between sputum and BAL sample collection, which may have influenced pathogen recovery and affected sensitivity and positivity rates. The potential impact of prior or ongoing antibiotic exposure was not assessed due to inconsistent documentation. Finally, the higher cost and resource requirements of BAL compared with sputum culture may limit its routine use in resource-constrained settings. Future prospective studies incorporating molecular diagnostics, controlling for antibiotic exposure, and evaluating patient outcomes are recommended to improve the clinical relevance and cost-effectiveness of sputum versus BAL cultures.

## Conclusions

This study highlights the complementary diagnostic roles of sputum and BAL cultures in identifying pathogens responsible for LRTIs. While sputum culture remains a valuable first-line diagnostic tool due to its high specificity and practicality, BAL offers superior sensitivity and a higher pathogen yield, particularly in clinically suspected cases with negative or inconclusive sputum results. Incorporating BAL selectively in such cases can enhance overall diagnostic accuracy and support more targeted antimicrobial management. Further large-scale, multicentric studies are warranted to validate these findings and optimize diagnostic strategies for pneumonia and other LRTIs.
